# Babassu (*Attalea speciosa*) Mesocarp Flour Extract Inhibits Lipid Peroxidation and Pro-Oxidant Enzymes: In Vitro and In Silico Evidence

**DOI:** 10.3390/foods15050945

**Published:** 2026-03-07

**Authors:** Caroline Chavier Pereira Santana, Fernanda Farias Costa, Jaqueline Daniele Santos Barros, Michelli Erica Souza Ferreira, Richard Pereira Dutra, Antônio Silva Machado, Aramys Silva Reis

**Affiliations:** 1 Laboratory of Pathophysiology and Therapeutic Investigation, Medical School, Center for Sciences of Imperatriz, Federal University of Maranhão, Imperatriz 65915-240, MA, Brazil; caroline.santana@discente.ufma.br (C.C.P.S.); fernanda.fc@discente.ufma.br (F.F.C.); michelli.ferreira@ufma.br (M.E.S.F.); 2 Graduate Program in Health and Technology, Center for Sciences of Imperatriz, Federal University of Maranhão, Imperatriz 65915-240, MA, Brazil; richard.dutra@ufma.br; 3 Laboratory of Natural Products Chemistry, Undergraduate Program in Natural Sciences, Center for Sciences of Imperatriz, Federal University of Maranhão, Imperatriz 65915-240, MA, Brazil; jaqueline.daniele@ufma.br; 4 Department of Health Sciences, State University of Tocantins, Augustinópolis 77960-000, TO, Brazil; antonio.m@untins.br

**Keywords:** *Attalea speciosa*, *Orbignya phalerata*, natural product, antioxidant, lipid peroxidation

## Abstract

(1) Background: Babassu (*Attalea speciosa)* mesocarp is a functional food rich in nutrients and phenolic compounds, offering antioxidant and metabolic benefits. However, its effects on lipid peroxidation and pro-oxidant enzymes remain poorly explored. (2) Methods: The antioxidant potential of a hydroalcoholic extract of babassu mesocarp (HEB) was assessed using DPPH radical scavenging and lipid peroxidation inhibition, measured by the TBARS assay. Cytotoxicity was assessed by the MTT assay. Molecular docking was conducted to investigate interactions between HEB-derived compounds and NADPH oxidase and xanthine oxidase. (3) Results: HEB showed dose-dependent antioxidant activity (IC_50_ = 4.734 µg/mL) and effectively inhibited lipid peroxidation (IC_50_ = 51.35 µg/mL), with no cytotoxic effects. In silico analyses suggested potential inhibition of pro-oxidant enzymes. (4) Conclusions: HEB exhibits a strong ability to inhibit lipid peroxidation and theoretical enzyme-inhibitory potential, supporting its use in functional foods and nutraceuticals.

## 1. Introduction

Babassu (*Attalea speciosa* syn. *Orbignya phalerata*) is one of the main non-timber forest products (NTFPs) of Brazilian sociobiodiversity and is a palm species widely distributed across the country, particularly in the transitional region between the Amazon and Cerrado biomes. Its exploitation represents a vital source of income and subsistence for socially and economically vulnerable communities [1]. The harvesting, cracking, and processing of babassu coconut is a traditional practice passed down through generations and carried out predominantly by women, known as babassu breakers. This activity represents an important supplementary source of income for their families, particularly during the agricultural off-season, and contributes to women’s economic autonomy [2]. According to data from the Brazilian Institute of Geography and Statistics (IBGE), 25,572 tons of babassu were produced in 2024, corresponding to a production value of BRL 71,647 [3]. This production primarily derives from extractivism and family farming, with the State of Maranhão, Brazil, standing out as the country’s leading producer.

The fruit of the babassu palm contains a lipid-rich kernel commonly used for babassu oil production, which is already a well-known commercial product. However, other parts of the fruit, considered byproducts of NTFPs, remain underused despite their nutritional and technological potential [4]. This potential has been explored in other NTFPs evaluated for application in functional foods. For example, mango, banana, pitaya, pineapple, and papaya peels have demonstrated significant antioxidant activity, both in their extracts and when incorporated as functional ingredients into food formulations [5].

Similarly, babassu mesocarp flour, one of the main underutilized byproducts, has emerged as a functional food ingredient with promising nutritional and technological applications. This byproduct can serve as a substitute for wheat flour in the preparation of different food products, including cakes [6], breads [7], cookies [8] pies and smoothies [9]. Notably, it represents an important nutritional source, supplying substantial amounts of carbohydrates, proteins, dietary fiber, minerals, and vitamins [6]. The antioxidant potential of babassu mesocarp has also been associated with photoprotection, an important application in cosmetic products [10]. The use of plant-based residues, such as babassu mesocarp flour, represents a sustainable alternative aligned with bioeconomy strategies and circular economy principles, as it reduces waste generation while adding economic value to underutilized materials [11]. Taken together, these findings highlight the potential of mesocarp utilization across different sectors, reinforcing its multifunctionality and sustainable application.

In addition to its nutritional composition, babassu mesocarp flour exhibits functional properties with potential health benefits. Experimental studies in mice have shown that its consumption, when combined with resistance training, can reduce retroperitoneal fat, highlighting its potential as an adjuvant for modulating body composition. Furthermore, total cholesterol and triglyceride levels decreased (*p* < 0.05) compared with non-supplemented groups, without adversely affecting blood glucose levels [12]. These effects may be associated with the high phenolic content in the mesocarp, which is known for its antioxidant activity [10,13,14]. The antioxidant capacity of babassu mesocarp has been [15,16], suggesting its potential to counteract oxidative stress and prevent the progression of chronic diseases related to free radical accumulation [17,18]. These combined metabolic and antioxidant effects support the value of babassu mesocarp flour as a multifunctional food ingredient with potential nutraceutical applications.

Although numerous studies have reported the antioxidant properties of babassu mesocarp using radical scavenging assays such as DPPH, ABTS, and FRAP [1,10,15,16]. These methods primarily measure generic electron or hydrogen donation capacity, offering limited insight into biologically relevant mechanisms of oxidative damage. Notably, the ability of babassu mesocarp compounds to prevent lipid peroxidation, a key pathway involved in cell membrane damage and disease progression [19], has not yet been evaluated. Addressing this gap is essential to better understand its functional potential, particularly in contexts where oxidative stress contributes to metabolic and chronic conditions [17,18]. Certain natural compounds can also modulate oxidative stress by neutralizing free radicals, inhibiting pro-oxidant enzymes, or enhancing endogenous antioxidant defenses [20,21,22].

In this context, the hydroalcoholic extract of babassu mesocarp may exert antioxidant activity not only through direct free radical scavenging and inhibition of oxidative processes, but also by modulating enzymes involved in the generation of reactive oxygen species. This broader mechanism would reinforce its potential as a multifunctional food ingredient with nutritional and metabolic relevance.

Enzymes such as xanthine oxidase and NADPH oxidase are major contributors to reactive oxygen species production in metabolic processes. Xanthine oxidase mediates the oxidation of hypoxanthine to uric acid, simultaneously generating free radicals [21,23], while NADPH oxidase facilitates superoxide production by transferring electrons derived from NADPH to molecular oxygen [24,25].

To investigate these mechanisms, computational in silico approaches provide a robust and economically viable strategy for predicting molecular interactions, pharmacokinetics, and bioactivity, allowing for the early identification of promising bioactive compounds [26,27,28]. These computational tools can complement experimental data and deepen our understanding of the antioxidant action of complex natural matrices.

This study evaluated the antioxidant capacity of babassu mesocarp extract by assessing its ability to inhibit lipid peroxidation and interact with key pro-oxidant enzymes using molecular docking. Additionally, in vitro cytotoxicity assays were conducted to provide a broader view of its functional and biological properties.

## 2. Materials and Methods

### 2.1. Babassu Mesocarp Flour

**Figure 1:**
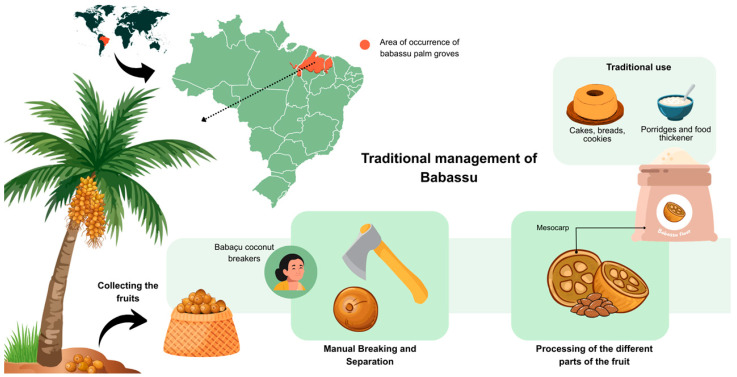
Distribution of babassu-producing areas in Brazil and traditional management practices.

The babassu mesocarp flour was acquired from the Interstate Cooperative of Women Babassu Coconut Breakers (*Cooperativa Interestadual das Mulheres Quebradeiras de Coco Babaçu*), São Luís, Maranhão, Brazil. This cooperative consists of women extractivists who harvest and process babassu fruits in the states of Pará, Maranhão, Tocantins, and Piauí (https://www.miqcb.org.br/ (accessed on 26 January 2026)). This study is registered in the Brazilian National System for the Management of Genetic Heritage and Associated Traditional Knowledge (SISGEN)—(*Sistema Nacional de Gestão do Patrimônio Genético e Conhecimentos Tradicionais Associados*), under code A7D3957. Figure 1 presents a map of the distribution of babassu-producing regions in Brazil, along with a schematic representation of the traditional management practices used for its exploitation.

### 2.2. Obtaining the Hydroalcoholic Extract of Babassu Mesocarp (HEB)

To prepare the extract, 200 g of babassu mesocarp flour was weighed, and 70% hydroethanolic solution (600 mL) was used as the solvent, at room temperature and without agitation. First, the flour was macerated at a 1:4 solvent ratio and left in the dark for 48 h. Afterward, the extract was filtered under vacuum and then concentrated in a rotary evaporator (model Q34432, Quimis, Sao Paulo, Brazil) at 45 °C. Finally, the extract was stored in a desiccator with silica, yielding a final yield of 3.76% [10].

This extract has already been characterized by a previous study conducted by our research group, in which the compounds present in this sample were identified using LC–MS analysis. The compounds identified are type B procyanidin dimer, (epi)catechin, type A procyanidin trimer, type A procyanidin dimer, quercetin-glucoside, quercetin and isorhamnetin, totaling seven compounds [10].

### 2.3. DPPH Radical Scavenging Assay

To assess antioxidant activity using the DPPH (2,2-diphenyl-1-picrylhydrazyl) radical assay, the hydroalcoholic extract of babassu mesocarp, previously diluted in methanol, was incorporated into a DPPH solution (Sigma-Aldrich, St. Louis, MO, USA at 40 µg/mL. Final concentrations of 2.5, 5, 10, and 20 µg/mL were obtained in the reaction mixture. The assay was conducted in the dark for 30 min, followed by absorbance measurement at 517 nm using a UV–Vis spectrophotometer (UV-340G, Gehaka, Sao Paulo, Brazil).

Ascorbic acid (2.5–10 µg/mL) served as the positive control. Radical scavenging activity was calculated according to the equation: AA (%) = 100 − [(A_sample_ − A_blank_) × 100/A_radical_], where A corresponds to absorbance.

The results were expressed as the IC_50_ value, defined as the concentration necessary to decrease the initial radical content by 50%. All experiments were carried out in triplicate [29].

### 2.4. Lipid Peroxidation Assay

Lipid peroxidation inhibition was assessed using the thiobarbituric acid reactive substances (TBARS) assay, based on a previously reported method with modifications [30]. For each assay tube, 250 µL of 5% egg yolk solution, 50 µL of babassu mesocarp extract prepared in PA methanol (Synth, Diadema, Brazil; 5000, 1000, 200, 40, and 8 µg/mL), 175 µL of distilled water, and 25 µL of Fenton’s reagent 0.07 M (freshly prepared as a 1:1 mixture of 0.14 M FeSO_4_ (Synth, Diadema, Brazil) and 0.14 M H_2_O_2_ (Synth, Diadema, Brazil) were combined. The mixture was then kept in the dark for 30 min to promote peroxidation. For the positive control, the same protocol was applied using Trolox (150 µg/mL; Sigma-Aldrich, St. Lois, MO, USA) instead of the extract to determine and compare antioxidant activity.

After incubation, 750 µL of 20% acetic acid (Synth, Brazil; pH 3.0), 750 µL of 0.8% TBA in 1.1% sodium dodecyl sulfate (SDS; Synth, Brazil), and 25 µL of 20% trichloroacetic acid (TCA; Synth, Brazil) were added to each tube, followed by vortex mixing and heating in a 95 °C water bath for 1 h. Once cooled, 3 mL of n-butanol (Synth, Brazil) were added to each tube, and the samples were centrifuged at 3000 rpm for 15 min.

Finally, the absorbance of the supernatant was recorded at 532 nm using a UV/Vis spectrophotometer (Gehaka UV-340G, Brazil).

### 2.5. In Vitro Cytotoxicity Assay

In vitro cytotoxicity was evaluated using the MTT (3-(4,5-dimethylthiazol-2-yl)-2,5-diphenyltetrazolium bromide) assay (Sigma-Aldrich, São Paulo, Brazil). The human fibroblast cell line GM07492A was seeded into 96-well plates at a density of 5 × 10^4^ cells per well in a final volume of 100 µL of DMEM (Gibco, Grand Island, NY, USA) supplemented with 10% fetal bovine serum (Gibco, Paisley, UK), 100 U/mL penicillin (Sigma-Aldrich, St. Lois, MO, USA), 100 µg/mL streptomycin (Gibco, Grand Island, NY, USA), and 2.5 µg/mL amphotericin B (Sigma-Aldrich, St. Lois, MO, USA). Plates were incubated overnight at 37 °C in a humidified atmosphere containing 5% CO_2_ to allow cell attachment.

Cells were then treated with babassu mesocarp hydroalcoholic extract at concentrations of 500, 100, 20, 4, and 0.8 µg/mL. Medium alone or extract at the corresponding concentrations served as the blank. Medium containing 0.7% ethanol was used as the negative control.

After 24 h of exposure, the medium was carefully removed and replaced with 90 µL of fresh DMEM, followed by the addition of 10 µL of MTT solution (5 mg/mL; Sigma-Aldrich, St. Louis, MO, USA). Plates were incubated in the dark for 3 h to allow formazan formation. Subsequently, the supernatant was discarded, and 100 µL of dimethyl sulfoxide (DMSO; Synth, Brazil) was added to each well to dissolve the formazan crystals. Plates were gently shaken for 5 min, and absorbance was measured at 550 nm using a microplate reader (BioTek, Winooski, VT, USA) [31]. All experiments were performed in triplicate

### 2.6. In Silico Analysis

#### 2.6.1. Protein Preparation

The three-dimensional structures of NADPH oxidase (NO) and xanthine oxidase (XO) were retrieved from the Protein Data Bank (PDB) [NADPH oxidase (NO): PDB ID 2CDU; resolution 1.80 Å; xanthine oxidase (XO): PDB ID 3NRZ; resolution 1.80 Å] [23]. Next, the protein structure was imported into SAMSON software platform (v. 2025, OneAngstrom, Grenoble, France).

Structural preparation was carried out using the native preparation module in SAMSON, which included: (i) addition of polar hydrogen atoms; (ii) removal of water molecules and co-crystallized ligands from the binding site; and (iii) geometry optimization and energy minimization using the Universal Force Field (UFF) [32] applying the steepest descent algorithm to eliminate steric clashes.

#### 2.6.2. Structural Preparation of the Ligands

The compounds identified in the hydroalcoholic extract of babassu mesocarp [10] were used as binders in the present study. All ligands were designed and converted to Structure Data File (SDF) format using ChemDraw v. 20.0 (CambridgeSoft Corporation, 2020, Cambridge, MA, USA). The 3D structures were imported into SAMSON (v. 2025), where explicit hydrogens were added. The ligand geometries were optimized via energy minimization with the Universal Force Field (UFF) in SAMSON to obtain the lowest-energy conformation before docking.

#### 2.6.3. Molecular Docking

Molecular docking analyses were conducted using the AutoDock Vina extension v.1.2.6 [33] integrated within the SAMSON platform. The search space (Grid Box) was defined visually in SAMSON, centered on the active site coordinates of the reference ligands (ADP and Hypoxanthine), ensuring sufficient dimensions to accommodate the ligands and allow rotational freedom. The docking calculations were executed with an exhaustiveness parameter set to 8. The resulting poses were ranked according to the Vina affinity score (kcal/mol). The best-scored conformations were visually analyzed in the SAMSON interface. The protocol was validated by redocking the standard ligands (ADP and Hypoxanthine) into their respective active sites to confirm the reproduction of the crystallographic binding mode.

### 2.7. Statistical Analysis

Statistical processing and graphical analyses were carried out using GraphPad Prism 8.4.3 (GraphPad Software, San Diego, CA, USA). The Shapiro–Wilk test was applied to assess the normality of variable distributions. The half-maximal inhibitory concentration (IC_50_) was determined by nonlinear regression analysis. Intergroup comparisons were performed using one-way ANOVA, followed by Tukey’s post hoc test, with statistical significance set at *p* < 0.05. Results are expressed as mean ± standard deviation.

## 3. Results

### 3.1. Evaluation of Antioxidant Activity by DPPH Radical Scavenging

**Figure 2:**
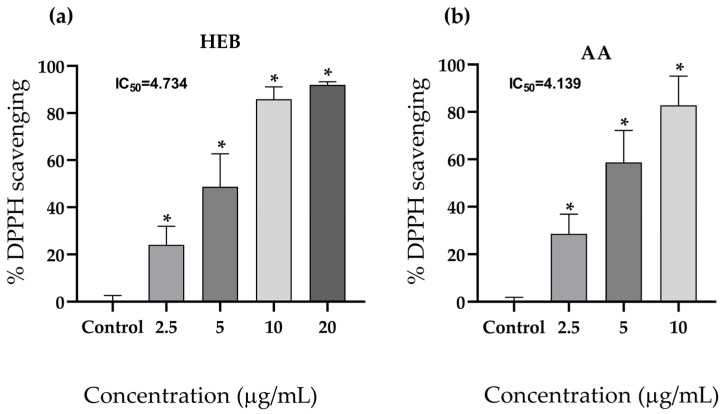
DPPH radical scavenging by babassu mesocarp hydroalcoholic extract (**a**) and ascorbic acid (**b**). The data represent the mean ± S.D. from six replicates of two independent experiments. The IC_50_ was calculated using nonlinear regression. HEB = babassu mesocarp hydroalcoholic extract. Differences between groups were determined by parametric ANOVA; * *p* < 0.05 compared with the control group. AA = ascorbic acid. IC_50_ = half-maximal inhibitory concentration.

The scavenging capacity of HEB against DPPH radicals was significantly higher at all tested concentrations (*p* < 0.001). Dose-dependent antioxidant activity was observed, with a IC_50_ of 4.734 (Figure 2a). The antioxidant performance of the extract, determined through the DPPH assay, was comparable to that of the positive control (ascorbic acid), which presented an IC_50_ of 4.139 (Figure 2b).

### 3.2. Lipid Peroxidation

**Figure 3:**
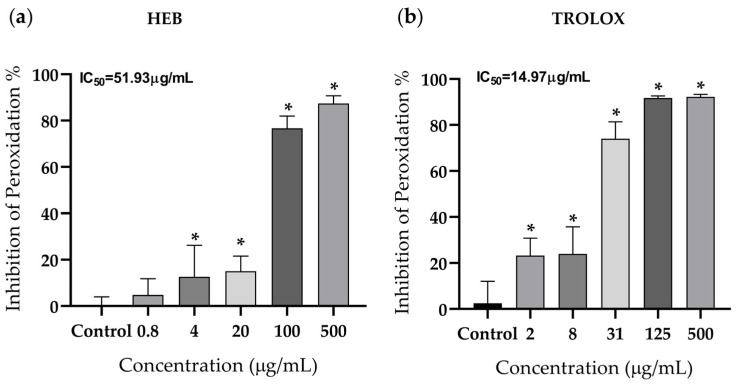
Lipid peroxidation inhibition by the babassu hydroalcoholic extract (**a**) and by the positive control (Trolox) (**b**). The data represent the mean ± S.D. from six replicates of two independent experiments. The IC_50_ was calculated using nonlinear regression. Differences between groups were determined by parametric ANOVA; * *p* < 0.05 compared with the control group. HEB = babassu mesocarp hydroalcoholic extract. IC_50_ = mean inhibitory concentration.

HEB significantly reduced lipid peroxidation at all evaluated concentrations (Figure 3a). Compared with the control group, lipid peroxide formation was significantly decreased at 500 and 100 µg/mL (*p* < 0.001), with an IC_50_ value of 51.35 µg/mL. Trolox, used as the positive control, showed dose-dependent inhibition of lipid peroxidation (IC_50_ = 14.97 µg/mL) (Figure 3b), with greater potency than the babassu extract.

### 3.3. Cytotoxicity

**Figure 4:**
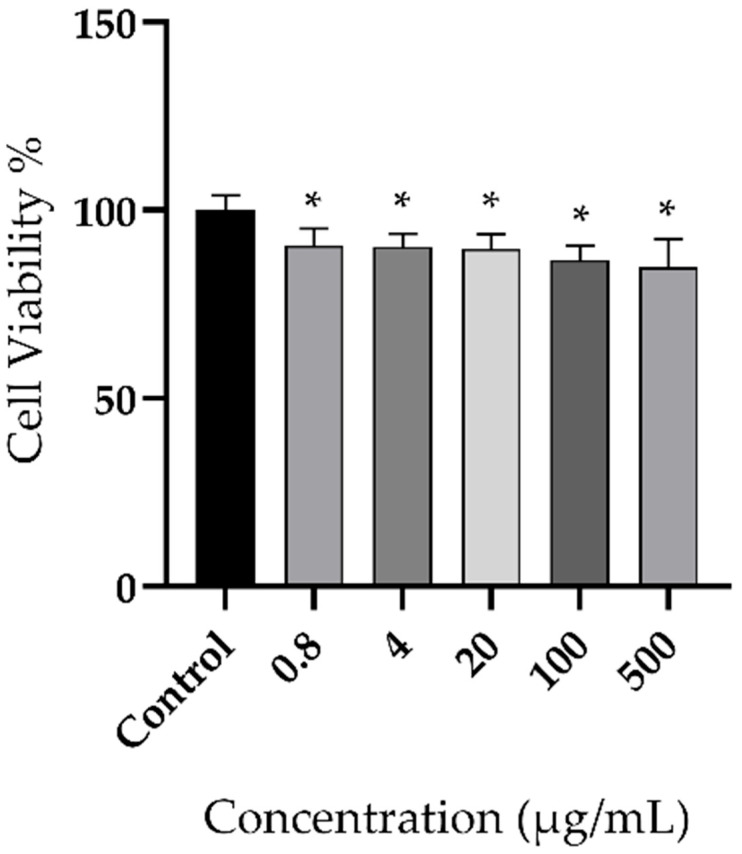
Effects of hydroalcoholic extracts of babassu mesocarp flour on human fibroblast cell line GM07492A viability. The data represent the mean ±SD of sextuplicate cultures from two independent experiments, and differences between groups were determined by parametric ANOVA; * *p* < 0.05 compared with the control group.

The cells treated with HEB maintained viability above 80% at all tested concentrations compared with untreated controls. These findings indicate no significant cytotoxic effects under the evaluated conditions (Figure 4).

### 3.4. Estudo In Silico

The molecular docking analysis included the compounds previously identified in HEB by LC–MS [10]. Overall, the constituents showed high affinity for the active sites of NADPH oxidase (NO) and xanthine oxidase (XO). Most compounds presented binding energies lower than those of the reference ligands, suggesting greater predicted affinity. The only exception was (epi)catechin against NO, which presented a slightly higher energy value than the standard. Among the identified metabolites, the type A procyanidin trimer showed the strongest binding potential for both enzymes, with free binding energies of −11.5 kcal/mol for NO and −10.4 kcal/mol for XO (Table 1).

Although the Type A procyanidin trimer exhibited superior interaction metrics (lower binding energy), its high molecular weight and abundance of hydroxyl groups may lead to nonspecific binding and steric hindrance, which often do not translate effectively to in vivo biological systems. Therefore, smaller molecules like quercetin-glycoside are more likely to yield favorable biological outcomes despite showing slightly lower theoretical binding energies compared to the trimer. Smaller molecular structures typically possess superior tissue permeability and transmembrane transport capabilities [34], favoring their bioavailability and interaction with intracellular targets.

**Figure 5:**
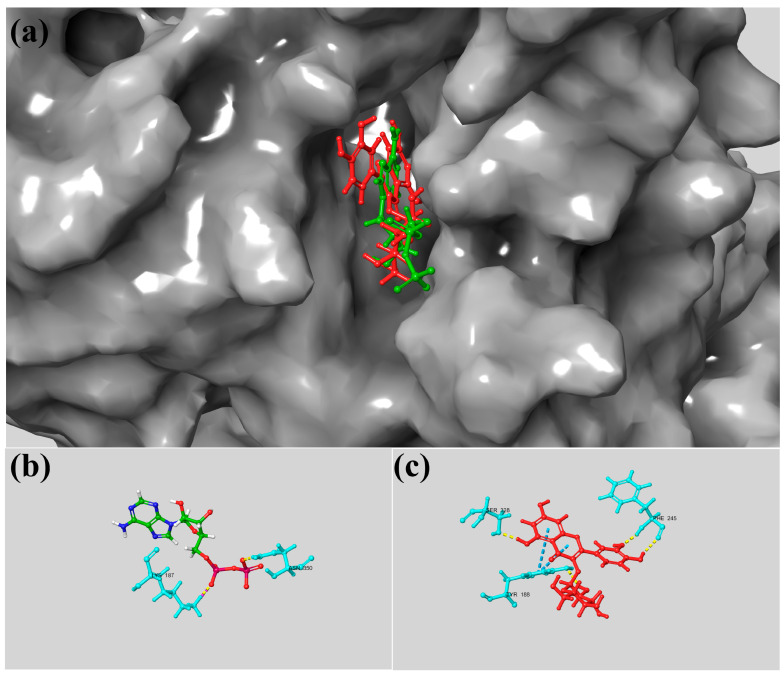
Detailed surface representation of the docking conformations of quercetin-glycoside (red) and ADP (green) within the active site of NADPH oxidase (NO; PDB ID: 2CDU; resolution: 1.80 Å). (**a**) Surface view of the NO catalytic pocket, showing the positioning of quercetin-glycoside and ADP. (**b**) Three-dimensional representation of the interactions established between ADP and the amino acid residues of the NO active site. (**c**) Three-dimensional representation of the interactions between quercetin-glycoside and the amino acid residues within the NO catalytic region. Dashed lines indicate hydrogen bonds, whereas solid lines denote van der Waals and other non-covalent interactions.

Regarding the interaction between quercetin-glycoside and NADPH oxidase, molecular docking analysis revealed that this flavonoid adopts a stable conformation within the enzyme’s active site. As shown in Figure 5a, quercetin-glycoside (red) is deeply embedded in the catalytic pocket, overlapping with the ADP binding region (green). Its binding affinity (−10.0 kcal/mol) surpassed that of the standard ligand, indicating a potent interaction. The 2D interaction diagram (Figure 5b) shows ADP interacting with key residues via hydrogen bonds and non-covalent contacts. In contrast, quercetin-glycoside establishes multiple stabilizing interactions, including hydrogen bonds with Tyr188, Phe245, and Ser328, alongside van der Waals forces with surrounding residues (Figure 5c). These contacts, combined with favorable hydrophobic and electrostatic interactions, underpin the high stability of the quercetin-glycoside–NADPH oxidase complex.

**Figure 6:**
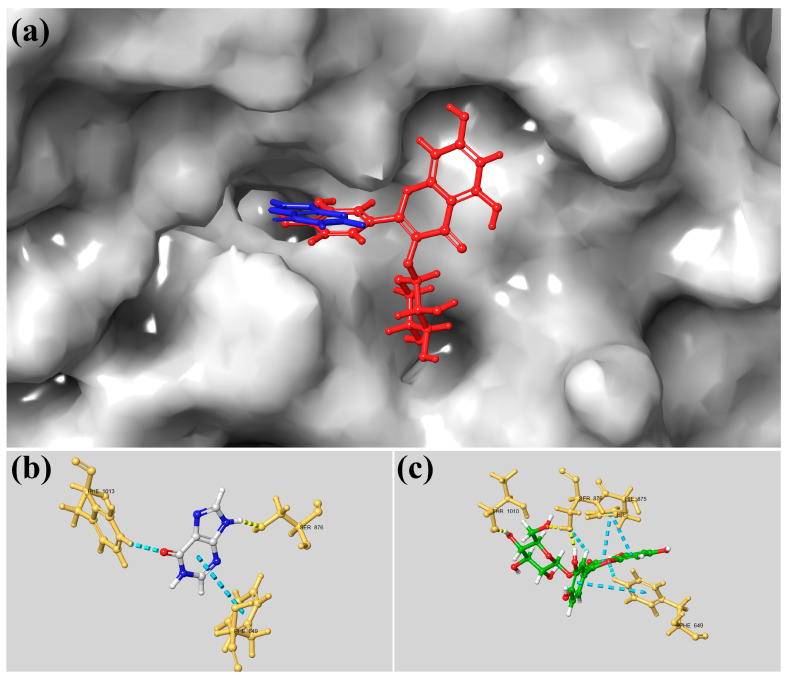
Detailed surface representation of the docking conformations of quercetin-glycoside (red) and hypoxanthine (blue) within the active site of xanthine oxidase (XO; PDB ID: 3NRZ, resolution: 1.80 Å). (**a**) Surface view of the XO catalytic pocket, showing the positioning of quercetin-glycoside and hypoxanthine. (**b**) Three-dimensional representation of the interactions established between hypoxanthine and the amino acid residues of the XO active site. (**c**) Three-dimensional representation of the interactions between quercetin-glycoside and the amino acid residues within the XO catalytic region. Dashed lines indicate hydrogen bonds, whereas solid lines denote van der Waals and other non-covalent interactions.

Similarly, for xanthine oxidase, the docking analysis demonstrated that quercetin-glycoside fits stably within the active site. Figure 6a illustrates the ligand (red) accommodated in the catalytic pocket, partially overlapping the hypoxanthine binding region (blue). The calculated binding affinity (−9.4 kcal/mol) suggests a strong inhibitory potential. While the hypoxanthine 2D diagram (Figure 6b) highlights interactions with key active site residues, quercetin-glycoside forms a robust network of stabilizing forces. These include hydrogen bonds with Ser876 and Thr1010, and π–π stacking interactions with Phe649 and His875, in addition to polar and hydrophobic contacts (Figure 6c). Collectively, these interactions contribute to the significant binding stability of quercetin-glycoside within the xanthine oxidase active site.

## 4. Discussion

This study demonstrates that the hydroalcoholic extract of babassu mesocarp flour exerts antioxidant activity not only through free radical scavenging and lipid peroxidation inhibition in vitro, but also through its interaction with pro-oxidant enzymes, as predicted by in silico analyses. DPPH assay revealed a concentration-dependent radical-scavenging response, indicating that antioxidant activity increased proportionally with extract concentration. This observation aligns with previous studies emphasizing the abundance of phenolic compounds in babassu mesocarp, which are widely recognized for their redox-regulating properties [10,14,35]. Furthermore, the antioxidant activity of babassu mesocarp extracts has also been demonstrated using complementary assays, such as ABTS and FRAP [1,10,15,16], supporting their involvement in multiple redox pathways and underscoring their potential application within the food sector.

Beyond radical scavenging, the suppression of lipid peroxidation observed in the TBARS assay reinforces the functional importance of the extract in lipid-containing systems. Lipid peroxidation represents a key process linked to cellular damage and the pathogenesis of oxidative stress–associated disorders, rendering its regulation particularly relevant for applications in food and nutraceutical contexts [36,37].

Notably, HEB exhibited a strong capacity to inhibit lipid peroxidation even at low concentrations (IC_50_ = 51.93 µg/mL), indicating a high antioxidant potency when compared with other natural products evaluated using similar experimental approaches. Extracts from *Phyllanthus fraternus* (IC_50_ = 1521 µg/mL) and *Tribulus terrestris* (IC_50_ = 377.66 µg/mL), although reported to exert protective effects against lipid peroxidation [30,38], require substantially higher concentrations to achieve comparable outcomes. This superior efficacy indicates the ability of babassu mesocarp flour to effectively reduce lipid-associated oxidative processes, supporting its relevance both for food quality preservation [4] and for the prevention of metabolic alterations associated with oxidative stress [12].

The inhibitory effect observed suggests that the extract components are capable not only of neutralizing free radicals but also of interrupting the propagation phase of lipid oxidation, which is particularly important in complex biological and food matrices. This effect is especially relevant considering that the TBARS assay differs from other antioxidant methods by actively generating reactive oxygen species during the assay, whereas assays such as DPPH and ABTS primarily evaluate the neutralization of pre-formed radicals [39,40].

The egg yolk lipid peroxidation model used in this study is a well-established, biologically relevant system for evaluating antioxidant activity in lipid-rich substrates [30]. However, as with any in vitro approach, it does not encompass the full metabolic, enzymatic, and physiological complexity of living organisms. Thus, while the model provides robust and reproducible evidence of lipid-protective activity, complementary cell-based assays and in vivo investigations will further expand the translational understanding of these findings [41].

Importantly, oxidative damage in biological systems may result from both radical-mediated reactions and enzymatic ROS generation, including pathways involving NADPH oxidase and xanthine oxidase [21,25,42]. Here, our in silico analyses demonstrate that phenolic compounds from babassu mesocarp exhibit high binding affinity for both enzymes, with binding energies surpassing those of reference standards, thereby supporting their potential as theoretical inhibitors. These results align with prior studies indicating that procyanidins and other phenolic constituents can modulate NADPH oxidase and xanthine oxidase activity, thereby mitigating oxidative stress through enzyme-mediated mechanisms [43,44,45]. However, these findings are preliminary theoretical evidence, and further in vitro enzymatic assays are necessary to confirm this activity. Although molecular docking results provide mechanistic insight into potential enzyme–ligand interactions, complementary kinetic and biochemical assays will be valuable for further substantiating these interactions under dynamic biological conditions [46].

NADPH oxidase plays a pivotal role in reactive oxygen species generation in endothelial and smooth muscle cells by mediating electron transfer from NAD(P)H to molecular oxygen, leading to the formation of superoxide anions (O_2_•^−^) [28]. These radicals are rapidly converted into hydrogen peroxide (H_2_O_2_), which can participate in Fenton-type reactions, leading to lipid peroxidation and membrane damage [25,47]. Furthermore, sustained NADPH oxidase activation is closely associated with apoptosis and localized membrane damage, as it interacts with membrane subunits and lipid components [48].

Similarly, xanthine oxidase contributes to oxidative stress by producing superoxide radicals and hydrogen peroxide during purine metabolism. Inhibition of this enzyme has been shown to attenuate lipid peroxidation, protein oxidation, and DNA damage [21]. Therefore, the capacity of HEB to interact with and potentially suppress both NADPH oxidase and xanthine oxidase indicates that, beyond free radical scavenging, this bioproduct may limit ROS generation at its primary source.

Furthermore, complementary evidence indicates that flavonoid-rich babassu mesocarp extracts retain antioxidant activity even after simulated gastrointestinal digestion, significantly reducing intracellular reactive oxygen species, restoring mitochondrial function, and modulating oxidative stress–related gene expression in HepG2 cells [14]. These findings strengthen the biological plausibility of the antioxidant effects observed in the present study and suggest that the bioactive compounds remain functional under physiologically relevant conditions.

Moreover, the safety profile of bioactive food-derived compounds represents a fundamental requirement. Therefore, cytotoxicity assays were performed using GM07492A human fibroblasts, revealing that HEB exhibited low cytotoxicity. These findings are in agreement with previous studies reporting the absence of cytotoxic effects of babassu extracts in different cell lines, including RAW 264.7 murine macrophages [10], and L929 fibroblasts [49], supporting their biocompatibility. Nevertheless, although these results support the safe use of HEB at the cellular level, further studies are required to comprehensively establish its safety. Further in vitro assays evaluating genotoxicity, oxidative balance across various cell types, and long-term exposure, along with rigorously designed in vivo and clinical studies, are necessary to establish safety, tolerability, and efficacy in humans. These investigations will provide critical evidence to support future application as a functional food ingredient.

Our results underscore the promising application of babassu mesocarp extract as a natural antioxidant ingredient. The ability to inhibit lipid peroxidation suggests promising use in functional foods, active packaging, or formulations designed to enhance oxidative stability. Supporting this notion, recent studies have demonstrated that babassu mesocarp–based materials can enhance the oxidative stability of food products and serve as biodegradable, bioactive components in food preservation systems. Future investigations should address the bioaccessibility, bioavailability, and metabolic fate of the extract’s bioactive compounds, and validate their efficacy in complex food matrices and in vivo models. Such approaches would further consolidate the functional value of babassu mesocarp extract and support its application as a sustainable, natural antioxidant source.

The findings presented in this study add to the expanding body of evidence supporting the valorization of babassu mesocarp, an underutilized agro-extractive by-product, as a source of bioactive compounds with notable antioxidant and lipid-protective properties. These results are consistent with contemporary trends in food science that emphasize sustainability, circular bioeconomy, and the development of functional ingredients from socio-biodiversity resources.

## 5. Conclusions

Our findings demonstrate, for the first time, that the hydroalcoholic extract of babassu mesocarp flour (HEB) exerts a pronounced inhibitory effect on lipid peroxidation and shows theoretical potential to inhibit pro-oxidative enzymes, as indicated by in silico analyses. Its antioxidant activity was further confirmed by effective free radical scavenging, suggesting complementary and synergistic mechanisms of action. Additionally, the low cytotoxicity observed in vitro supports the preliminary safety of HEB for food and nutraceutical applications.

These findings suggest that the babassu mesocarp extract has promising functional properties and potential nutraceutical applications. Although further enzymatic validation and in vivo and clinical investigations are warranted to strengthen its mechanistic and translational relevance, the current evidence demonstrates consistent antioxidant performance across chemical, biological, and lipid-based systems. Overall, HEB represents a promising candidate for the development of functional foods and nutritional supplements, aligning biological efficacy with sustainability and the valorization of underutilized agrofood by-products.

## Figures and Tables

**Table 1:** Values of free binding energies obtained by molecular docking of HEB compounds.

NADPH Oxidase (NO)		Xanthine Oxidase (XO)	
Compost	^ΔG^bind (kcal/mol)	Compost	^ΔG^bind (Kcal/mol)
ADP (standard)	−6.3	Hypoxhantine (standard)	−3.1
Type A procyanidin trimer	−11.5	Type A procyanidin trimer	−10.4
Quercetin-glucoside	−10.0	Type B procyanidin dimer	−9.5
Type A procyanidin dimer	−9.1	Quercetin-glucoside	−9.4
Type B procyanidin dimer	−8.8	Type A procyanidin dimer	−8.8
Quercetin	−7.6	Quercetin	−7.0
Isorhamnetin	−6.7	(epi) Catechin	−6.9
(epi) Catechin	−5.9	Isorhamnetin	−5.3

## Data Availability

The original contributions presented in this study are included in the article. Further inquiries can be directed at the corresponding author.

## Abbreviations

The following abbreviations are used in this manuscript:

HEB	Hydroalcoholic extract of babassu mesocarp
DPPH	2,2-difenil-1-picril-hidrazil
NADPH	Nicotinamide adenine dinucleotide hydrogenated phosphate
ROS	Reactive oxygen species
IC_50_	Mean inhibitory concentration
